# The prevention of home-cage grid climbing affects muscle strength in mice

**DOI:** 10.1038/s41598-022-19713-4

**Published:** 2022-09-10

**Authors:** Hiroshi Ueno, Yu Takahashi, Shinji Murakami, Kenta Wani, Tetsuji Miyazaki, Yosuke Matsumoto, Motoi Okamoto, Takeshi Ishihara

**Affiliations:** 1 Department of Medical Technology, Kawasaki University of Medical Welfare, Kurashiki, Okayama 288, Matsushima701-0193 Japan; 2 Department of Psychiatry, Kawasaki Medical School, Kurashiki, 701-0192 Japan; 3 Department of Neuropsychiatry, Graduate School of Medicine, Dentistry and Pharmaceutical Sciences, Okayama University, Okayama, 700-8558 Japan; 4 Department of Medical Technology, Graduate School of Health Sciences, Okayama University, Okayama, 700-8558 Japan

**Keywords:** Neuroscience, Zoology, Neurology

## Abstract

Experimenters and treatment methods are the major contributors to data variability in behavioral neuroscience. However, home cage characteristics are likely associated with data variability. Mice housed in breeding cages spontaneously exhibit behavioral patterns such as biting into the wire grid and climbing on the grid lid. We aimed to clarify the effect of covering the stainless steel wire grid lid in commonly used home cage with Plexiglas to prevent climbing on muscle strength in mice. Furthermore, we investigated the effects of climbing prevention on activity and anxiety-like behavior, and the impact of climbing prevention during the postnatal development period and adulthood on muscle strength. Muscle strength, anxiety-like behavior, and locomotor activity were assessed by a battery of tests (wire hang, suspension, grip strength, rotarod, elevated-plus maze, and open field tests). Mice prevented from climbing the wire grid during postnatal development displayed lower muscle strength than those able to climb. Moreover, mice prevented from climbing for 3 weeks following maturity had weakened muscles. The muscle strength was decreased with 3 weeks of climbing prevention in even 1-year-old mice. In summary, the stainless steel wire grid in the home cage contributed to the development and maintenance of muscle strength in mice.

## Introduction

Currently, laboratory mice play a central role in the study of animal models of human behavioral disorders^1^. Several laboratories worldwide use identical genetically defined mouse strains and mutant mice to address complex behavioral questions. The use of mice in biomedical research can be traced back to the 1600 s; since then, this species has contributed to numerous scientific discoveries and advances in basic biological and pharmaceutical research^2^. It is necessary to clarify the appropriate treatment, handling method, and breeding method of laboratory mice to successfully transfer the obtained results to human experiments.

Furthermore, there is growing interest in the reproducibility of mouse behavioral phenotypes^3^. In recent years, the "reproducibility crisis" has strongly influenced science. Experimenters^4,5^ and processing methods^6^ may be the major contributors to data variability in behavioral neuroscience. However, the mouse home cage environment also contributes to this variability^7^.

The breeding conditions for laboratory mice are principally based on economics (minimum use of space, equipment, and labor), ergonomics (easy to handle, animal visibility), hygiene (easy to disinfect), and standardization^8,9^. Mice are usually housed in transparent “shoe-box” cages containing bedding, food, and water. In home cages, mice perform natural, spontaneous, and often complex behaviors, such as play, care, socializing, and nesting^10^. Healthy rodents display voluntary selective behaviors, sometimes referred to as elective behaviors, and are motivated to perform them^10,11^. Mice housed in standard home cages spontaneously exhibit several behavioral patterns, including bar gnawing, bar swiveling, and bar jumping, all of which are involved in climbing the grid cage lid^12^. The physical and psychological characteristics of mice may be affected by the degree of performance of these behaviors in the home cage. The inhibition of climbing behavior in the home cage from the 3rd to 7th week of life affects anxiety-like behavior and activity in female mice^13^. Climbing the stainless steel lid of the home cage is thought to affect the increase in, and maintenance of, muscle strength and the motor performance of mice. Commercially available mouse home cages have different lids that either allow or prevent climbing behavior. However, many articles inadequately describe the characteristics of home cage used.

Generally, depressive behavior, aggression, activity, and anxiety-like behavior of mice are measured by a series of behavioral tests to determine experimental effects^14^. The behavioral test battery is important for detecting abnormal behavior in mice^15–18^. Further, it is necessary to understand the role of muscle strength and motor ability of mice in this series of behavioral tests, as these characteristics considerably affect the amount of activity and anxiety-like behavior.

The wire hang test is an easy and inexpensive way to assess muscle performance in small rodents. This test uses a wire grid system to non-invasively measure the ability of mice to exhibit sustained limb tension against the body weight, and assesses multiple aspects of athletic performance, including grip strength, endurance, and physical coordination. Particularly, the wire hang test is widely used to determine the natural course of neuromuscular diseases, demonstrate neuromuscular disorders and the lack of coordination, and study the effects of genetic or pharmacological treatments on skeletal muscle function^19–21^.

Hand grips are universally used to assess the physical performance of older adults. Mice are widely used as a model for aging studies^22^. The mouse grip strength test is similar to that used in humans, in that it assesses the ability to grip a device with the foot, and is non-invasive and easy to perform.

In the present study, we aimed to clarify the effect of preventing grid-climbing activity on muscle strength in mice by covering the stainless steel wire grid lid in a commonly used home cage with Plexiglas (i.e. the mouse was not allowed to climb and hang on the lid). Furthermore, we investigated the effects of climbing prevention on activity and anxiety-like behavior, and the impact of climbing prevention during the postnatal development period and adulthood on muscle strength. In the present study, we focused on the reproducibility of behavioral tests under different climbing conditions. In behavioral testing, mice around 10 weeks old are typically used; therefore, we conducted behavioral experiments at 10 weeks of age.

## Materials and methods

### Animals

All animal experiments were performed in accordance with the ARRIVE guidelines (http://www.nc3rs.org.uk/arrive-guidelines) and the U.S. National Institutes of Health (NIH) Guide for the Care and Use of Laboratory Animals (NIH Publication No. 80-23, revised in 1996), and were approved by the Committee for Animal Experiments at the Kawasaki Medical School Advanced Research Center. All efforts were made to minimize the number of animals used and their suffering. C57BL/6 N male mice were purchased from Jackson Laboratory (Kanagawa, Japan) and housed in cages (four to five animals per cage), with food and water provided ad libitum under a 12-h light/dark cycle at 23–26 °C.

### Behavioral tests

All behavioral tests were conducted in behavioral testing rooms, between 09:00 and 16:00, during the light phase of the circadian cycle. Behavioral tests were separated by at least 1 day of no testing. On each testing day, mice were tested in a random order. Following each test, the equipment was cleaned with 70% ethanol and super-hypochlorous water to prevent bias caused by olfactory cues. We performed the behavioral tests in naïve mice. Behavioral tests were performed in the order described below. At the end of the study, the mice were sacrificed by CO_2_ inhalation^23^.

### Wire grid-climbing prevention

**Figure 1:**
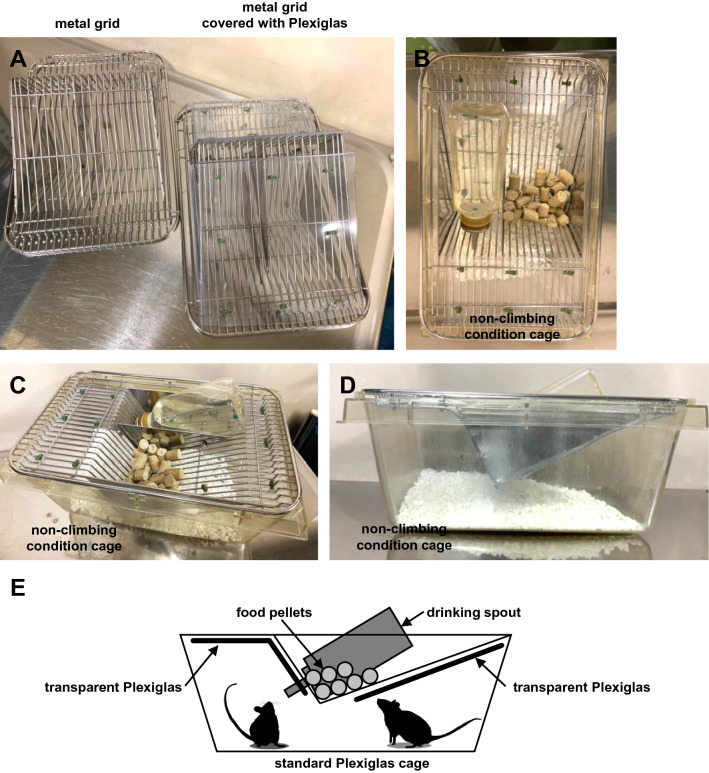
The cages used in the climbing and non-climbing conditions. (**A)** Sample picture of a metal grid lid and one covered with Plexiglas. (**B**) Sample picture of a non-climbing condition cage (from above). (**C**) Sample picture of a non-climbing condition cage (from diagonally above). (**D**) Sample picture of a non-climbing condition cage (from the side). (**E**) Schematic diagram of the non-climbing condition cage.

In the climbing condition (i.e. control condition), mice were housed in a standard plastic cage (20 × 30 × 20 cm) with a stainless steel wire grid lid (Fig. 1A). In the non-climbing condition, mice were housed in the same standard plastic cage as the control mice, but to prevent wire grid climbing, the stainless steel wire grid lid was covered with Plexiglas (Fig. 1A–E).

### Effects of grid-climbing prevention during postnatal development

**Figure 2:**
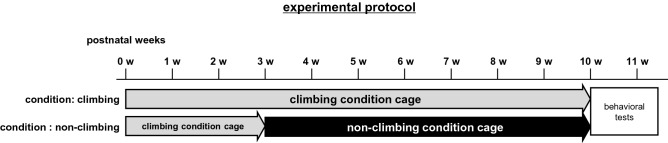
Experimental protocol. Experimental time schedules: mice in the non-climbing group are housed in cages with wire grid lids covered with Plexiglas from 3 to 10 weeks of age. Subsequently, behavioral tests were performed.

To investigate the effects of climbing prevention during postnatal development, we randomly assigned 3-week-old male mice into one of two housing conditions; namely, the climbing condition (n = 15) and the non-climbing condition (n = 15). Mice were housed in their respective cages until they were 10 weeks old (Fig. 2).

### Effects of grid-climbing prevention in adulthood

To investigate the effects of climbing prevention after maturity, we randomly assigned 3-week-old male mice into one of three housing conditions as follows: (1) the climbing condition, in which mice were housed in the climbing cage until they were 13 weeks old (n = 10); (2) the non-climbing [3–10 weeks] condition, in which mice were housed in the non-climbing cage until they were 10 weeks old (n = 15); and (3) the non-climbing [10–13 weeks] condition, in which mice were housed in the non-climbing cage until they were 13 weeks old (n = 15).

### Effects of grid-climbing prevention in middle age

To investigate the effects of climbing prevention in middle age, we randomly assigned 1-year-old male mice into one of two housing conditions as follows: (1) the climbing condition, in which mice were housed in the climbing cage for 3 weeks (n = 5); and (2) the non-climbing condition, in which mice were housed in the non-climbing cage for 3 weeks (n = 10).

### Effects of promoted-grid-climbing in adulthood

To investigate the effects of promoted climbing in adulthood, a breeding cage with food pellets at a height of 15 cm was prepared by covering the lower part of the wire grid with Plexiglas (promoted-climbing cage; Fig. 10A). We randomly assigned 10-week-old male mice into one of two housing conditions as follows: (1) the climbing condition, in which mice were housed in the climbing cage for 3 weeks (n = 10); and (2) the promoted-climbing condition, in which mice were housed in the promoted climbing cage for 3 weeks (n = 10).

### Wire hang test

For the wire hang test, mice were placed on a wire mesh that was subsequently inverted and gently waved, forcing the mice to grip the wire. We recorded the latency to fall. A wire hang test apparatus (O’Hara & Co., Tokyo, Japan) was used for this test.

### Wire suspension test

For the wire suspension test, we placed a 2-mm-thick wire parallel to the floor and secured it approximately 40 cm above a layer of bedding. We let the mice grasp the wire with their forepaws only; we lowered the hindlimbs so that mice hung from the wire only by their forepaws. We recorded the latency to fall^24^.

### Grip strength test

We used a grip strength meter to assess the forelimb grip strength. Mice were lifted and held by their tail such that their forepaws could grasp a wire grid; subsequently, they were gently pulled backwards until they released the grid. We recorded the peak force applied by the mouse’s forelimbs in centinewtons (cNs).

### Rotarod test

We assessed motor coordination and balance using the rotarod test^25^. This test used an accelerating rotarod (RTR-M5; Melquest, Toyama, Japan). Mice were placed on a rotating drum (3.9-cm diameter) and the time they were able to maintain balance on this rod was measure. The speed of the rotarod was accelerated from 4 to 40 rpm over a period of 5 min. The intertrial interval for this test was 20 min. All mice were subjected to the test without any pre-test training.

### Hot plate test

We used the hot plate test to evaluate nociception (sensitivity to a painful stimulus)^26^. The mice were placed on a hot plate at 55.0 °C ± 0.3 °C, and the latency to the first paw response (foot shake or paw lick) was recorded^27^. A latency period of 30 s was defined as complete analgesia and used as the cut-off time to prevent tissue injuries.

### Elevated plus-maze test

Anxiety-like behavior was examined using the elevated plus-maze test^28,29^. The apparatus consisted of two each opposing open and closed arms (each 8 × 25 cm), with 30-cm-high transparent plastic walls. The arms were constructed from white acrylic plates, and the maze was elevated to a height of 40 cm^23^. The center of the apparatus was illuminated at 100 lx. Each mouse was placed in the central square of the maze facing one of the closed arms, and was allowed to freely explore the four arms for 6 min. The mice were video recorded, and the distance travelled (m), number of entries into open arms, and time spent in the open arms (s) were analyzed using video-tracking software (ANY-MAZE, Stoelting Co., Wood Dale, IL, USA).

### Open field test

We used the open field test to examine exploratory behavior, anxiety-like behavior, and general locomotor activity^17,30^. Each mouse was placed in the center of an apparatus comprising a square area surrounded by white acrylic walls (45 × 45 × 40 cm)^23^. We recorded the total distance travelled (m), number of entries into the central area, and time spent in the central area (s). The central area was defined as the middle 20 × 20 cm area of the field. The test chamber was illuminated at 100 lx. Data were collected over 20 min. Data analysis was performed using ANY-MAZE software.

### Wire grid climbing measurement

For measurements of wire grid climbing, mice were acclimated to the single housing environment, and their behavior was monitored from 14:00 to 11:00 the day after acclimation. Images were captured via a video camera, and we measured the number of episodes and amount of time climbing on the wire grid. We used 4-week- (n = 4), 10-week- (n = 4), and 1-year-old (n = 4) mice for this test.

### Statistical analyses

Statistical analyses were performed using SPSS software (IBM Corp, Tokyo, Japan). Data were analyzed using the one-way analyses of variance (ANOVA), followed by Tukey’s test, or two-way repeated-measures ANOVA, followed by Fisher’s least significant difference test. *P*-values < 0.05 were considered statistically significant. All data are presented as box plots or the mean ± standard error.

### Ethics approval and consent to participate

All animal experiments were performed in accordance with the U.S. National Institutes of Health (NIH) Guide for the Care and Use of Laboratory Animals (NIH Publication No. 80-23, revised in 1996) and approved by the Committee for Animal Experiments at the Kawasaki Medical School Advanced Research Center.

## Results

### Effects of grid-climbing prevention on physical characteristics

**Figure 3:**
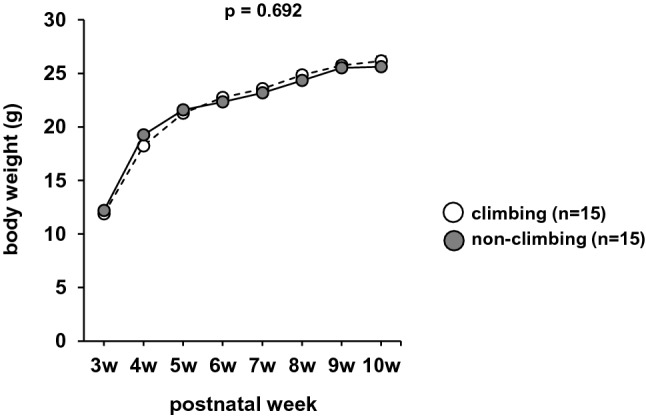
The effect of grid-climbing prevention on body weight. Body weight gain (in grams) following weaning (at 3 weeks of age), until 10 weeks of age. Data are presented as the mean ± standard errors. Statistical significance is represented by asterisks: *p < 0.05, ^+^*p* < 0.1. *P*-values were calculated using the two-way repeated-measures analysis of variance. Climbing: n = 15; non-climbing: n = 15.

**Figure 4:**
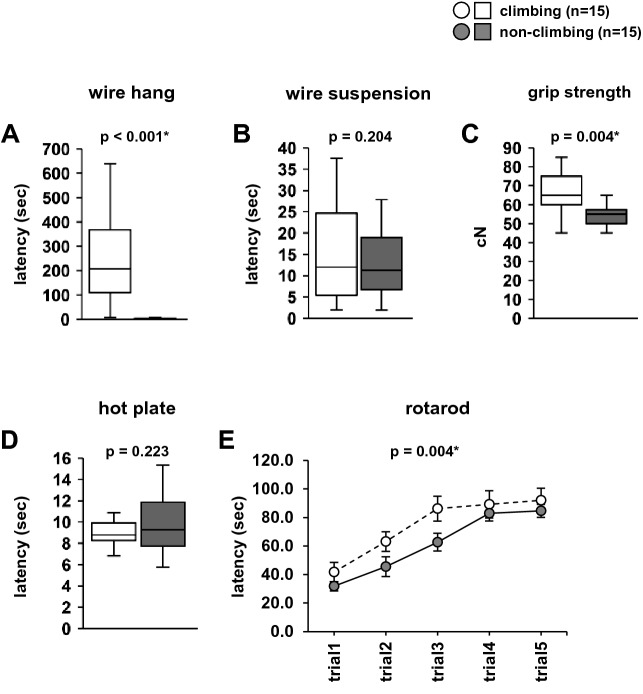
The effect of grid-climbing prevention on physical characteristics. The latency to fall in the wire hang test (**A**), latency to fall in the wire suspension test (**B**), grip strength (**C**), latency to the first paw response in the hot plate test (**D**), and latency to fall in the rotarod test (**E**). Data are presented as box plots (**A**–**D**) or mean ± standard error (**E**). Statistical significance is represented by asterisks: *p < 0.05, ^+^*p* < 0.1. *P*-values were calculated using the one-way analysis of variance (ANOVA) (**A**–**D**) or two-way repeated-measures ANOVA (**E**). (**A**–**E**) climbing: n = 15; non-climbing: n = 15.

There were no significant differences in body weight gain between the climbing and non-climbing mice (Fig. 3, *F*1,224 = 0.158, *p* = 0.692). In the wire hang test, the latency to fall was significantly lower in the non-climbing mice than in the climbing mice (Fig. 4A, climbing mice: 241.847 ± 45.234, non-climbing mice: 3.607 ± 0.689, *F*1,28 = 27.734, *p* < 0.001; Sup Video S1, S2). In the wire suspension test, there was no significant difference between climbing and non-climbing mice in the latency to fall (Fig. 4B, F1,28 = 1.693, *p* = 0.204). Mice in the non-climbing condition frequently exhibited the behavior of holding the wire in their axillae (Sup Videos S3, S4). Grip strength was significantly lower in the non-climbing mice than in the climbing mice (Fig. 4C, F1,28 = 9.872, *p* = 0.004). In the hot plate test, there were no significant differences between climbing and non-climbing mice (Fig. 4D, F1,28 = 1.553, *p* = 0.223). In the rotarod test, the latency to fall was significantly lower in the ton-climbing mice than in the climbing mice (Fig. 4E, F1,140 = 8.734, p = 0.004).

### Effects of grid-climbing prevention on the elevated plus-maze test

**Figure 5:**
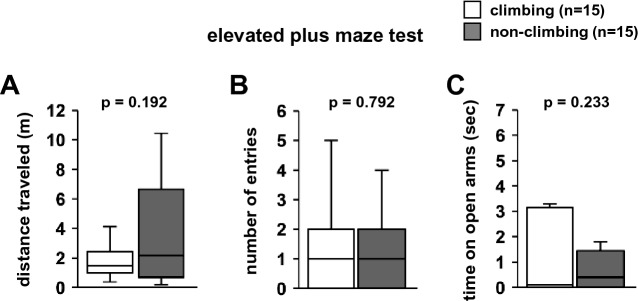
The effect of grid-climbing prevention on performance in the elevated plus-maze test. Elevated plus-maze test: the total distance travelled (**A**), number of entries into open arms (**B**), and time spent in open arms (**C**). Data are presented as box plots. Statistical significance is represented by asterisks: *p < 0.05, ^+^*p* < 0.1. *P*-values were calculated using the one-way analysis of variance (**A**-**C**). (**A**–**C**) climbing: n = 15; non-climbing: n = 15.

In the elevated plus-maze test, there were no significant differences between climbing and non-climbing mice in the total distance travelled (Fig. 5A, F1,28 = 1.789, *p* = 0.192), total number of entries in open arms (Fig. 5B, F1,28 = 0.071, *p* = 0.792), and time spent in the open arms (Fig. 5C, F1,28 = 1.485, *p* = 0.233).

### Effects of grid-climbing prevention on the open field test

**Figure 6:**
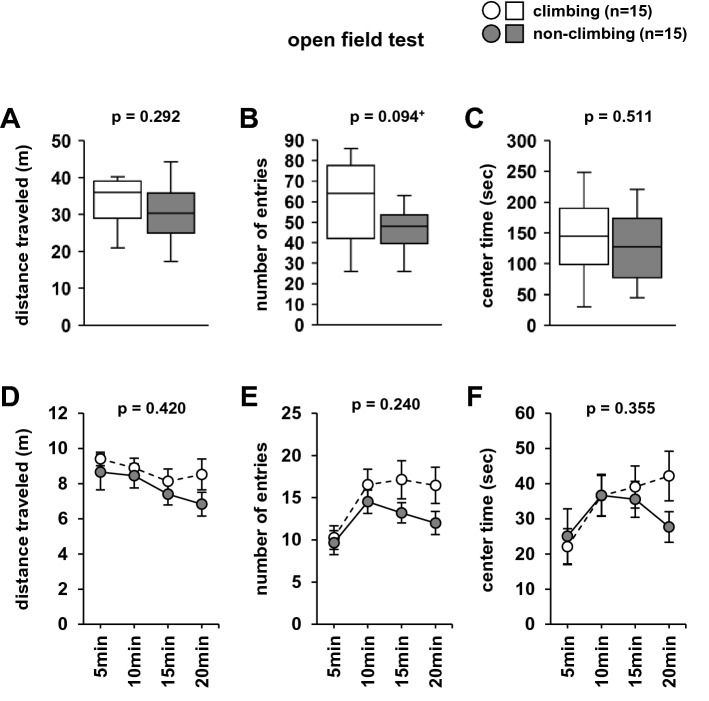
The effect of grid-climbing prevention on the performance in the open field test. Graphs displaying the total distance travelled (**A**), total number of entries into the central area (**B**), total time spent in the central area (**C**), distance travelled in each 5 min period (**D**), number of entries into the central area in each period (**E**), and time spent in the central area in each period (**F**). Data are presented as box plots (**A**–**C**) or mean ± standard error (**D**–**F**). Statistical significance is represented by asterisks: *p < 0.05, ^+^*p* < 0.1. *P*-values were calculated using the one-way analysis of variance (ANOVA) (**A**–**C**) or two-way repeated-measures ANOVA (**E**). (**A**–**E**) climbing: n = 15; non-climbing: n = 15.

In the open-field test, there were no significant differences between climbing and non-climbing mice in the distance travelled (Fig. 6A, F1,28 = 1.152, *p* = 0.292, Fig. 6D, F1,112 = 0.973, *p* = 0.420), number of entries into the central area (Fig. 6B, F1,28 = 3.003, *p* = 0.094, Fig. 6E, F1,112 = 1.444, *p* = 0.240), and center time (Fig. 6C, F1,28 = 0.441, *p* = 0.511, Fig. 6F, F1,112 = 1.097, *p* = 0.355).

### Effect of grid-climbing prevention in adulthood

**Figure 7:**
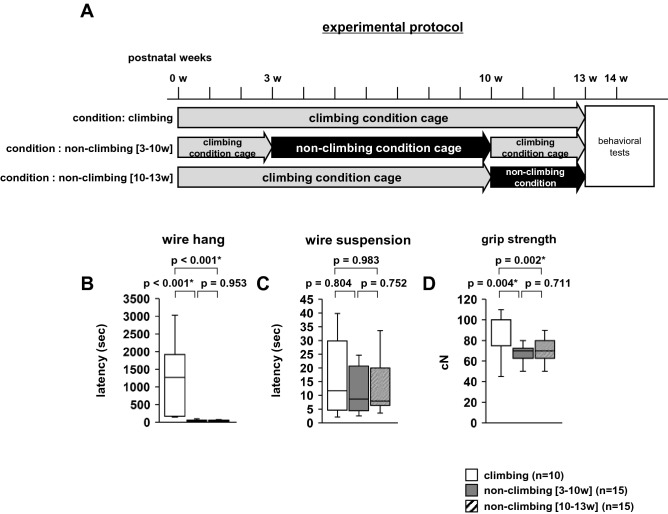
The effect of grid-climbing prevention on physical characteristics in adulthood. (**A**) Experimental time schedules: non-climbing [3–10 weeks] mice are housed in cages with wire grid lids covered with Plexiglas from 3 to 10 weeks of age and housed in standard cages from 10 to 13 weeks of age. Non-climbing [10–13 weeks] mice are housed in standard cages until 10 weeks of age and housed in cages with wire grid lids covered with Plexiglas from 10 to 13 weeks of age. Subsequently, behavioral tests were performed. The latency to fall in the wire hang test (**B**) and wire suspension test (**C**), and grip strength test (**D**). Data are presented as box plots (**B**–**D**). Statistical significance is represented by asterisks: *p < 0.05, ^+^*p* < 0.1. *P*-values were calculated using the one-way analysis of variance (**B**–**D**). (**B**–**D**) climbing: n = 15; non-climbing: n = 15.

Subsequently, we examined the effects of grid-climbing prevention in mature mice (non-climbing [10–13 weeks] vs control, Fig. 7A). Moreover, we examined the effects of resuming gird climbing following maturity (non-climbing [3–10 weeks] vs control, Fig. 7A). Following 3 weeks of treatment, we examined their neuromuscular strength. In the wire hang test, the latency to fall was significantly lower in the non-climbing [3–10 weeks] mice than in the climbing mice (Fig. 7B, climbing mice: 1194.774 ± 326.614, non-climbing [3–10 weeks] mice: 46.536 ± 9.626, non-climbing [10–13 weeks] mice: 57.454 ± 11.030, *F*_2,37_ = 18.787, *p* < 0.001). Furthermore, the latency to fall was significantly lower in the non-climbing [10–13 weeks] mice than in the climbing mice. In the wire hang test, there were no significant differences between non-climbing [3–10 weeks] and non-climbing [10–13 weeks] mice. In the wire suspension test, there were no differences in the latency to fall among the three conditions (Fig. 7C, F2,37 = 0.060, *p* = 0.942). Grip strength was significantly lower in the non-climbing [3–10 weeks] mice than in the climbing mice (Fig. 7D, F2,37 = 8.356, *p* = 0.001), and in the non-climbing [10–13 weeks] mice than in the climbing mice. There were no significant differences in grip strength between non-climbing [3–10 weeks] and non-climbing [10–13 weeks] mice.

### Wire grid climbing in the home cage

**Figure 8:**
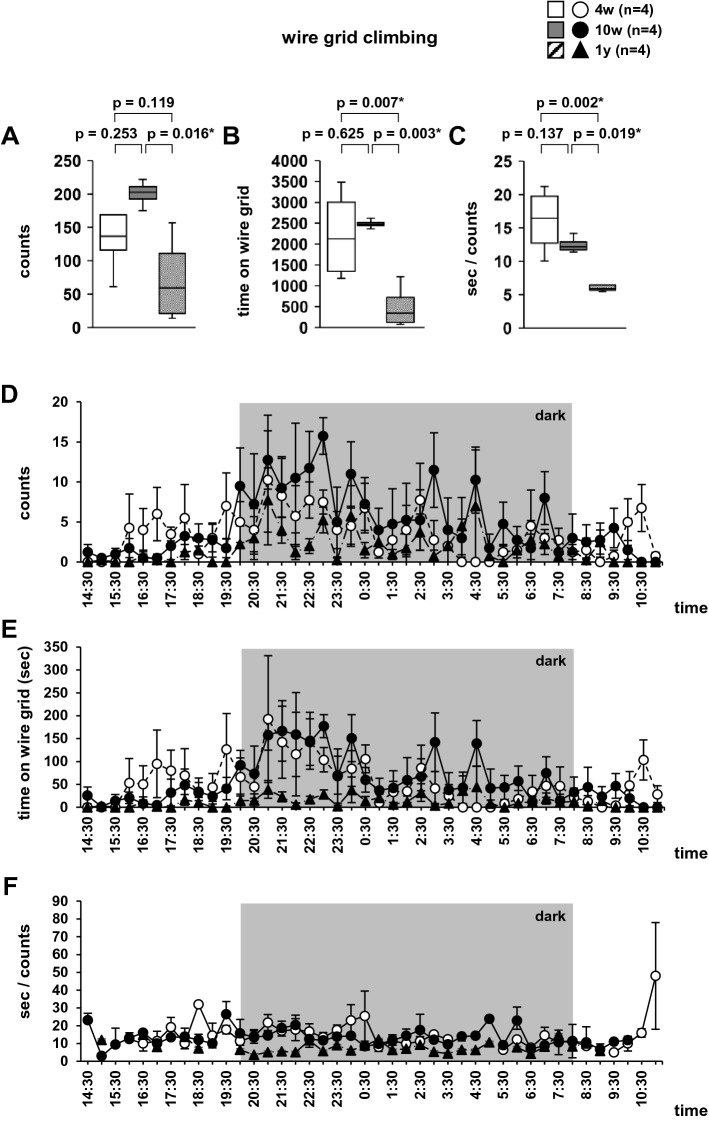
Wire grid climbing in the home cage. Graphs displaying the total number of episodes of grid climbing (**A**), total time spent grid climbing (**B**), mean time per grid-climbing episode (**C**), number of episodes of grid climbing in each 30 min period (**D**), time spent grid climbing in each period (**E**), and mean time per grid-climbing episode in each period (**F**). Data are presented as box plots (**A**–**C**) or mean ± standard error (**D**–**F**). Statistical significance is represented by asterisks: *p < 0.05, ^+^*p* < 0.1. *P*-values were calculated using the one-way analysis of variance (**A**–**C**). (**A**–**E**) climbing: n = 4; non-climbing: n = 4.

Additionally, we examined grid climbing behavior in the home cage in 4-week-, 10-week-, and 1-year-old mice. There was no significant difference between 4-week- and 10-week-old mice in the total number of gird-climbing episodes (Fig. 8A, F2,9 = 4.379, *p* = 0.047). However, the number of grid-climbing episodes was significantly lower in 1-year-old mice than in 10-week-old mice. Additionally, the total time spent grid climbing was significantly lower in 1-year-old mice than in both 4-week- and 10-week-old mice (Fig. 8B, F2,9 = 9.162, *p* = 0.007). There was no significant difference between 4-week- and 10-week-old mice in the total time spent grid climbing. The mean time per grid-climbing episode was significantly lower in 1-year-old mice in both 4-week- and 10-week-old mice (Fig. 8C, F2,9  = 10.209, *p* = 0.005). There was no significant difference between 4-week- and 10-week-old mice in the mean time per grid-climbing episode. We plotted graphs for the number of gird-climbing episodes in each 30 min period (Fig. 8D), time spent grid climbing during each period (Fig. 8E), and mean time grid climbing during each period (Fig. 8F). High climbing activity was observed in the early dark phase.

### Effect of grid-climbing prevention in middle-aged mice

**Figure 9:**
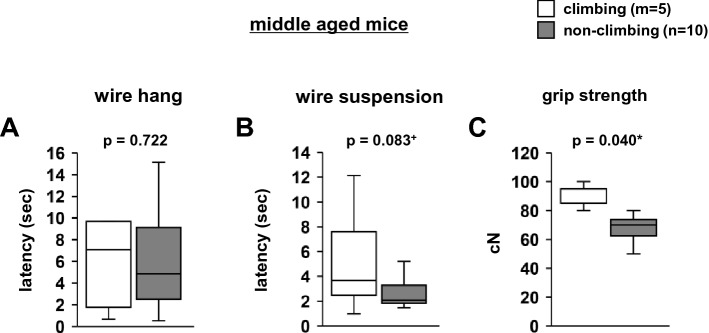
The effect of grid-climbing prevention on physical characteristics in middle age. The latency to fall in the wire hang test (**A**) and wire suspension test (**B**), and grip strength test (**C**). Data are presented as box plots (**A**–**C**). Statistical significance is represented by asterisks: *p < 0.05, ^+^*p* < 0.1. *P*-values were calculated using the one-way analysis of variance (**A**–**C**). (**A**–**C**) climbing: n = 15; non-climbing: n = 15.

We examined the effects of grid-climbing prevention in weight-matched (within 2 g) 1-year-old mice. Following climbing prevention for 3 weeks, we examined their neuromuscular strength. There were no significant differences between climbing and non-climbing 1-year-old mice in the wire hang test (Fig. 9A, F1,13 = 0.132, *p* = 0.722) and wire suspension test (Fig. 9B, F1,13 = 3.527, *p* = 0.083). Grip strength was significantly lower in 1-year-old non-climbing mice than in 1-year-old climbing mice (Fig. 9C, F1,13 = 5.181, *p* = 0.040).

### Effects of promoted grid climbing in adulthood

**Figure 10:**
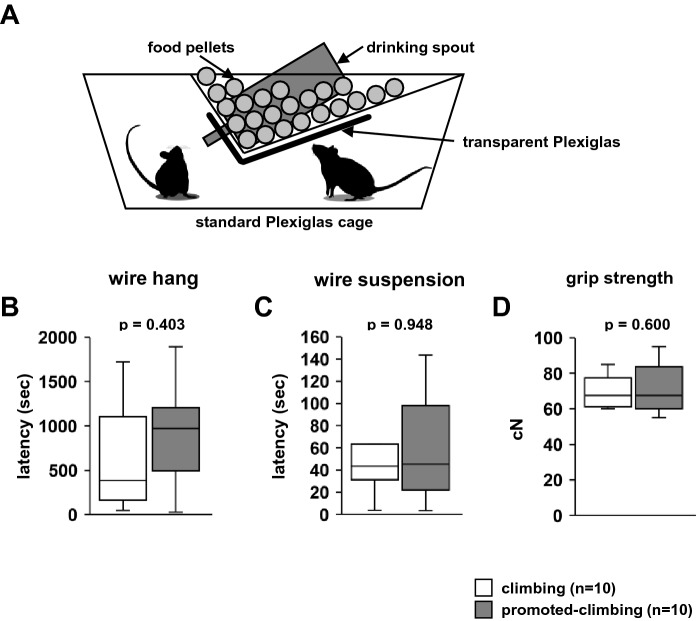
The effect of promoted-climbing on physical characteristics in adulthood. (**A**) A schematic diagram of the promoted-climbing condition cage. The latency to fall in the wire hang test (**B**) and wire suspension test (**C**), and grip strength test (**D**). Data are presented as box plots (**B**–**D**). *Statistical significance is represented by asterisks: *p < 0.05, ^+^*p* < 0.1. *P*-values were calculated using the one-way analysis of variance (**B**–**D**). (**B**–**D**) climbing: n = 10; non-climbing: n = 10.

Subsequently, we examined the effects of promoted grid climbing in mature mice (Fig. 10A). We raised the position of the food pellets in the home cage to facilitate grid climbing. Following 3 weeks of treatment, we examined their neuromuscular strength. There were no differences between climbing and promoted-climbing mice in the latency to fall in the wire hang test (Fig. 10B, F1,18 = 0.734, *p* = 0.403) and wire suspension test (Fig. 10C, F1,18 = 0.004, *p* = 0.948), and in grip strength test (Fig. 10D, F1,18 = 0.286, *p* = 0.600).

## Discussion

The purpose of the present study was to investigate the effect of preventing climbing on the stainless steel wire bars in the lid of commonly used cages on the muscle strength of mice. We found that stainless steel wire lids in the home cage contributed to the development and maintenance of muscle strength in mice.

Mice that were prevented from climbing the grid lid in their home cage from post-weaning to maturity did not demonstrate a difference in body weight gain compared to those that were allowed to climb. We had predicted weight loss with climbing prevention because of stress. Chronic stress reportedly reduces body weight in mice^31,32^. However, the present results suggest that grid-climbing prevention was not a chronic stress for the mice. Furthermore, there were no changes in activity level with grid-climbing prevention, as indicated by the elevated plus-maze and open field test results. Considered together, these results suggest that the energy level in the home cage did not change despite the prevention of grid climbing in the home cage. In order to further clarify the presence or absence of stress with climbing prevention, it is necessary to measure stress hormones and the amount of food consumption in future studies.

Mice housed in breeding cages voluntarily perform several behavioral patterns, including bar gnawing, bar swiveling, and bar jumping, all of which are involved in climbing the grid cage lid^12^. These interactions with the steel bars present in the cage lid represent key elements of behavior in the home cage of laboratory mice^33^; however, there are no reports on their association with muscle strength. Researchers have reported that the forelimb grip strength in mice is maximal at 4 months, decreases at 7 months, and is stable until 2 years of age^34^. In contrast, the hindlimb grip strength significantly increases from 4 to 7 months, and decreases at 18 months of age. Researchers have not investigated the relationship between grip strength and the lid characteristics of the home cage. In the present study, mice that were prevented from climbing the wire grid lid from post-weaning to maturity demonstrated a marked decrease in muscle strength compared to that in mice that were allowed to climb, as assessed by the wire hang and grip strength tests. In the wire suspension test, mice that were prevented from climbing the grid lid did not hold the wire in their forelimbs, but instead held it with their armpits. This behavior indicated that there was no habit of grabbing the wire with the forelimbs or that the forelimbs were significantly weak. Accordingly, the lid grid of the home cage promoted the development of muscle strength in developing mice. This necessitates further research to investigate the essential muscle development of mice and the period of development.

Motor coordination in mice has traditionally been assessed by the rotarod test^35,36^. In this test, mice must remain upright and walk forward to avoid falling. It is a sensitive, objective, and quantifiable test. Rodents with motor dysfunction fall off the rod faster than those with normal motor function^34^. In the present study, mice that were prevented from climbing the grid lid following weaning displayed decreased athletic performance in the rotarod test. Thus, lid grid climbing in the home cage provided an opportunity for developing mice to cultivate athletic performance.

Mice that were prevented from grid climbing and then subsequently housed in cages allowing grid climbing for 3 weeks did not have similar strength to control mice. However, we did not compare the strength of these mice initially to those with continued grid climbing prevention; accordingly, it is unknown whether muscle strength was partially restored. It is possible that these mice did not develop the habit of climbing the lid grid, thus further research is necessary to clarify the findings.

All of the mice (regardless of condition) tended to be less active in the elevated plus maze test than in the open field test, suggesting that they had anxiety for heights. It is also possible that the mice behaved differently in low light. Following weaning, mice that were prevented from grid climbing did not demonstrate changes in their activity or anxiety-like behavior in the elevated plus maze and open field tests. Preventing climbing behavior in the home cage for only 4 weeks following birth has been shown to cause behavioral abnormalities in female mice^13^. However, the prevention of climbing behavior did not cause anxiety-like behavior in the male mice used in the present study. This suggests that grid-climbing prevention may not affect the psychological behavior of male mice. Further research is required to directly compare the effect of grid-climbing prevention on anxiety-like behavior in male and female mice.

In the hot plate test, mice prevented from grid climbing did not show a change in sensitivity to temperature. Hot plate tests are commonly used to assess heat pain susceptibility^37^. The present results demonstrate that grid-climbing prevention does not exert an effect on the development of nociception in mice.

In the present study, the number and time of grid-climbing episodes depended on the age of the mouse. Furthermore, we found that 1-year-old mice also exhibited effects of grid-climbing prevention on muscle strength. Climbing behavior in the home cage is known to vary with age, sex, and tension^38^. In a previous study of 1-, 2-, 3-, 4-, 5-, and 6-month-old mice, the maximum climbing time was observed at 2 months of age^38^, consistent with the present results. A decline in climbing behavior with aging is associated with a decline in overall activity and exploratory drive with aging^39,40^. The present findings demonstrated that climbing behavior in the home cage is required for the development and maintenance of muscle strength and motor ability in mice, even in 1-year-old mice. Further research is needed to determine whether increasing exploratory behavior in the home cage increases climbing behavior and maintains muscle strength.

In the wire hang and grip strength tests, mature mice displayed a decrease in muscle strength after grid-climbing prevention for 3 weeks compared to that in control mice. Peak climbing behavior is observed at approximately 2 months following birth. As mice exhibit climbing behavior throughout their lives^39,40^, muscle strength can be maintained by climbing the grid lid in the home cage. Climbing prevention in 10-week-old mice has been reported to result in decreased muscle strength in grip strength tests^41^. The observed decrease in muscle strength indicated that muscles were weakened by the prevention of grid climbing. Furthermore, 3 weeks of prevention was sufficient to induce muscle weakness. Further research is warranted to determine whether the duration of prevention and age of mice exert different effects on muscle weakness. Additionally further studies are needed to obtain detailed data on muscle mass and muscle atrophy.

In 1-year-old mice, preventing grid climbing for 3 weeks did not result in any change in the wire hang and wire suspension tests; nonetheless, it resulted in a decrease in grip strength. These results indicate that middle-aged mice prevented from grid climbing have decreased forelimb grip strength. The grip strength of forelimbs in mice remains stable after 7 months of age; however, that of the hindlimbs decreases at 18 months following birth^32^. In the present study, 1-year-old mice exhibited climbing behavior in their home cage. This climbing behavior in the home cage was associated with the maintenance of forelimb grip strength in mice at up to 12 months of age. To prevent mouse habituation to the grip strength test, we did not perform the grip strength test prior to grid-climbing prevention. Therefore, it is possible that there was a pre-existing difference in the grip strength in the mice.

In the present study, mice housed in home cages with elevated food to promote climbing did not display increased muscle strength. Mice frequently hang in their home cage^38^. It is unclear if the number and time of grid-climbing episodes were increased in the home cage environment used in this experiment; however, it did not lead to further muscle strengthening. Thus, grid climbing in normal breeding cages is sufficient for maintaining muscle strength in mice.

Grid bar-related activities have been described as stereotypes owing to their repetitive and invariant nature; however, this activity should merely be considered as a form of exercise or behavioral habit^42,43^. On the hand, grid bar-related activities may represent an attempt to leave the home cage and explore the external environment^12^. The role of grid bar-related activities in representing purposeless or functional behavior remains debatable. The present experimental results did not address this argument. However, the climbing behavior of mice in the home cage can be effectively used for pain assays^38^. The present findings revealed that the climbing behavior with respect to the grid bar promoted the development of muscle strength in mice, and was related to its maintenance.

The early post-weaning period is sensitive to environmental effects on the rodent brain^44^. Changes in housing conditions during this period exert long-term effects on both brain and behavior^45^. Environmental enrichment is a widely applied term and includes an experimental paradigm in which intensive environmental enrichment strategies are used to investigate the impact of complex environments^46^. This environmental enrichment causes changes in mouse behavior^47^, emotions^48,49^, physiology^50^, and neurobiology^51,52^, compared to that with normal simple cages^53^. However, the direction and magnitude of the effect of environmental enrichment depends on the type and combination of enrichment^54^, mice strain^55^, sex^56^, time and duration of enrichment^57^, and parameters studied^58^. Researchers have not yet comprehensively investigated the effects of the shape and material of the lid in the home cage on mouse behavior. Mice climb the lid and roam through the home cage^59^. Moreover, they gnaw on the wire grid on the lid^60^. The present findings suggest that the mice climbed the wire grid lid to develop and maintain their muscle strength. Therefore, the wire grid lid of the home cage also contributes to environmental enrichment.

Muscle weakness is an important phenotype of several diseases associated with movement disorders and increased mortality. The forces generated by a muscle are largely determined by the size of the muscle, type of fiber, and coupling process of excitation and contraction. The strength of the limbs of experimental mice can be evaluated using a commercially available grip strength meter. This test is a widely used non-invasive method, specifically used to assess the strength of the forelimbs and/or hindlimbs of mice to assess effects of disease (e.g., cancer, dystrophy) or drug treatment on skeletal muscle^61,62^. Wire climbing is ideal for measuring the coordination and endurance of mice muscles^63,64^. Moreover, it has been applied to assess the grip strength, balance, and endurance in mice following cerebral infarction^65^. However, the relationship between the lid of the home cage and muscle strength has not been previously reported. The present findings indicate that previous reports on mouse muscle weakness may be affected by the shape of the lid and the exploratory behavior of mice in their home cage. For example, mice may improve their muscle strength by frequently climbing the grid lid in the home cage, despite genetic modification to reduce their muscle strength. Researchers should pay attention to the cage lid upon conducting experiments on mouse muscle strength. In addition, a description of the lid of the home cage is required in the "Materials and methods" section.

The present results were obtained using only male mice. Climbing behavior in the home cage depends on age and sex^38^, and its effect on the muscle strength of mice may differ. This warrants further research using female mice.

## Conclusion

The wire grid on the lid of the home cage contributed to the development and maintenance of mouse muscle strength. Researchers should pay attention to the shape and material of the mouse cage lid.

## Supplementary Information

Supplementary Video 1.

Supplementary Video 2.

Supplementary Video 3.

Supplementary Video 4.

Supplementary Information 1.

## Data Availability

The dataset is available on reasonable request from the corresponding author.

## Abbreviation

ANOVA: Analysis of variance
