# Supplementary material for: The prevention of home-cage grid climbing affects muscle strength in mice
Source: Sci Rep. 2022 Sep 10;12:15263. doi: 10.1038/s41598-022-19713-4 (PMC9464241; doi:10.1038/s41598-022-19713-4)
Supplement: Supplementary file 5 — Supplementary Information 1. [file 41598_2022_19713_MOESM5_ESM.docx]

1. ORIGINAL RESEARCH ARTICLE

**The Prevention of Home-Cage Grid Climbing Affects Muscle Strength in Mice**

**Hiroshi Ueno^1,^*, Yu Takahashi^2^, Shinji Murakami^2^, Kenta Wani^2^, Tetsuji Miyazaki^2^, Yosuke Matsumoto^3^, Motoi Okamoto^4^, Takeshi Ishihara^2^**

1. 1 Department of Medical Technology, Kawasaki University of Medical Welfare, Okayama, 701-0193, Japan
2. 2 Department of Psychiatry, Kawasaki Medical School, Kurashiki, 701-0192, Japan
3. 3 Department of Neuropsychiatry, Graduate School of Medicine, Dentistry and Pharmaceutical Sciences, Okayama University, Okayama, 700-8558, Japan
4. 4 Department of Medical Technology, Graduate School of Health Sciences, Okayama University, Okayama, 700-8558, Japan

**Author Emails**

Hiroshi Ueno, PhD. E-mail: dhe422007@s.okayama-u.ac.jp

Yu Takahashi, MD. E-mail: yuuu.takahashi@gmail.com

Shinji Murakami, PhD, MD. E-mail: muraka@med.kawasaki-m.ac.jp

Kenta Wani, PhD, MD. E-mail: k-wani@med.kawasaki-m.ac.jp

Tetsuji Miyazaki, PhD, MD. E-mail: t_miyazaki@med.kawasaki-m.ac.jp

Yosuke Matsumoto, PhD, MD. E-mail: ymatsumoto@cc.okayama-u.ac.jp

Motoi Okamoto, PhD, MD. E-mail: mokamoto@md.okayama-u.ac.jp

Takeshi Ishihara, PhD, MD. E-mail: t-ishihara@med.kawasaki-m.ac.jp

***Corresponding author:**

1. Hiroshi Ueno, PhD.
2. Address: Department of Medical Technology, Kawasaki University of Medical Welfare, 288, Matsushima, Kurashiki, Okayama, 701-0193, Japan
3. Phone: +81-86-462-1111, Fax: +81-86-462-1193
4. E-mail address: dhe422007@s.okayama-u.ac.jp

**Supplementary Figure Legends**

**Supplementary Video 1** Wire hang test in climbing mice

This is an 8 × speed movie showing the performance behavior of two mice.

**Supplementary Video 2** Wire hang test in non-climbing mice

This is a movie showing the performance behavior of six mice.

**Supplementary Video 3** Wire suspension test in climbing mice

This is a movie showing the performance behavior of five mice.

**Supplementary Video 4** Wire suspension test in non-climbing mice

This is a movie showing the performance behavior of five mice.
